# The Evolution and Appearance of C3 Duplications in Fish Originate an Exclusive Teleost *c3* Gene Form with Anti-Inflammatory Activity

**DOI:** 10.1371/journal.pone.0099673

**Published:** 2014-06-13

**Authors:** Gabriel Forn-Cuní, Edimara S. Reis, Sonia Dios, David Posada, John D. Lambris, Antonio Figueras, Beatriz Novoa

**Affiliations:** 1 Institute of Marine Research, Consejo Superior de Investigaciones Científicas (CSIC), Vigo, Spain; 2 Department of Pathology and Laboratory Medicine, School of Medicine, University of Pennsylvania, Philadelphia, Pennsylvania, United States of America; 3 Department of Biochemistry, Genetics and Immunology, Universidad de Vigo, Vigo, Spain; Karlsruhe Institute of Technology, Germany

## Abstract

The complement system acts as a first line of defense and promotes organism homeostasis by modulating the fates of diverse physiological processes. Multiple copies of component genes have been previously identified in fish, suggesting a key role for this system in aquatic organisms. Herein, we confirm the presence of three different previously reported complement *c3* genes (*c3.1*, *c3.2*, *c3.3*) and identify five additional *c3* genes (*c3.4*, *c3.5*, *c3.6*, *c3.7*, *c3.8*) in the zebrafish genome. Additionally, we evaluate the mRNA expression levels of the different *c3* genes during ontogeny and in different tissues under steady-state and inflammatory conditions. Furthermore, while reconciling the phylogenetic tree with the fish species tree, we uncovered an event of *c3* duplication common to all teleost fishes that gave rise to an exclusive *c3* paralog (*c3.7* and *c3.8*). These paralogs showed a distinct ability to regulate neutrophil migration in response to injury compared with the other *c3* genes and may play a role in maintaining the balance between inflammatory and homeostatic processes in zebrafish.

## Introduction

The zebrafish (*Danio rerio*) has been increasingly recognized in biomedical research as a valuable model with which to study vertebrate development, hematopoiesis and immunity [1]. As free-living organisms from early embryonic life stages, fish are highly dependent on their innate immune system for survival [2]. Among the diverse group of cells and proteins that comprise innate immunity, the complement system is considered an essential first-line defense mechanism not only in fish but also in other vertebrates and invertebrates [3].

The complement system is recognized as an intricate set of plasma and cell-surface proteins that interact with each other in an organized cascade, leading to system activation and the release of biologically active proteins. The complement system modulates the fates of diverse physiological processes, from inflammation and pathogen opsonization and clearance to hematopoiesis, tissue regeneration and lipid metabolism [4]. In vertebrates, complement can be activated by three distinct pathways: the classic, alternative and lectin, all of which converge at the C3 level with consequent cleavage of the C3 and C5 proteins and the generation of anaphylatoxins and other biologically active fragments.

C3 is an approximately 185-kDa protein that comprises 13 different domains organized into two chains (alpha and beta) that are connected by a disulfide bond [5]. C3 is one of the most abundant proteins in the plasma (approximately 1.6 mg/ml in humans) and, through its diverse domains, can interact with a wide variety of plasma and cellular proteins [6].

A body of evidence from evolutionary genetics studies has indicated the presence of the *C3* gene in organisms that existed before the divergence between Cnidaria and Bilateralia [7]. Since then, the *C3* gene has maintained an evolutionary equilibrium and is highly conserved among species, likely due to its importance in immunity and homeostasis mechanisms [4]. Positive evolutionary pressure on *C3* seems to be particularly pronounced in fish, in which *C3* gene duplications have been characterized in a variety of species, such as trout [8], common carp [9], medaka fish [10] and zebrafish [11]. Interestingly, previous studies have indicated that the multiple *C3* genes present in a single species are associated with the recognition of different fish pathogens, thus enlarging the spectrum of pathogen-associated molecular patterns (PAMPs) that the complement system can recognize and respond to [8].

Herein, we confirm the presence of three different *c3* genes and identify five additional *c3* genes in the zebrafish genome. Furthermore, we propose that an early duplication event occurred at the base of the teleost fish clade. Most importantly, we show the differential abilities of C3 paralogs to regulate cytokine production and neutrophil migration upon injury, indicating a dual role for complement in the inflammation/regeneration processes in zebrafish.

## Materials and Methods

### Phylogenetic analysis

An exhaustive *BLAST* search [12] was performed against the *Danio rerio* full genome (version Zv9) with the available human *C3* and zebrafish *c3* sequences that were retrieved from the public NCBI nucleotide database (http://www.ncbi.nlm.nih.gov/nucleotide). Similarities and identities between the corresponding protein sequences were calculated with MatGAT 2.02 [13]. Structural characterizations were investigated with the NCBI online Conserved Domain Database (CDD) [14].

Additional fish C3 and C5 protein sequences were retrieved from published genomes with the Ensembl Genome Browser, version 69 [15] (Table 1). The sequence alignment was performed with the MAFFT online server according to the E-INS-i strategy [16]. Ambiguously aligned columns were pruned with Gblocks 0.91b [17]. The best-fit model of amino acid replacement was selected according to the Akaike Information Criterion (AIC) [18] with ProtTest 3.2 [19]. The *c3*–*c5* gene family tree was estimated with jPrime 0.2.0 [20], in which 4 independent MCMC runs, each consisting of 1,000,000 iterations, were sampled once every 200 iterations. After discarding the first 500 samples for each run as burn-in, the final gene tree was obtained as a weighted consensus majority-rule tree from the 4 runs with MrBayes 3.2.1 [21], [22].

**Table 1:** *Ensembl* Protein IDs used in the study.

Species	Gene	Ensembl Gene ID	Species	Gene	Ensembl Gene ID
***Petromyzon marinus***	*c3.1*	ENSPMAP00000009280	***Latimeria chalumnae***	*c3.1*	ENSLACP00000018716
	*c3.2*	ENSPMAP00000004086		*c3.2*	ENSLACP00000018425
***Danio rerio***	*c3.1*	ENSDARP00000052687		*c5*	ENSLACP00000014054
	*c3.2*	ENSDARP00000108724	***Gasterosteus aculeatus***	*c3.1*	ENSGACP00000001657
	*c3.3*	ENSDARP00000052682		*c3.2*	ENSGACP00000024919
	*c3.4*	ENSDARP00000117916		*c3.3*	ENSGACP00000024929
	*c3.5*	ENSDARP00000120827*		*c3.4*	ENSGACP00000024774
	*c3.6*	ENSDARP00000064201		*c3.5*	ENSGACP00000026212*
	*c3.7*	ENSDARP00000118396		*c5*	ENSGACP00000019556*
	*c3.8*	ENSDARP00000098698	***Oreochromis niloticus***	*c3.1*	ENSONIP00000020727
	*c5*	ENSDARP00000088095		*c3.2*	ENSONIP00000020671
***Gadus morhua***	*c3.1*	ENSGMOP00000019326		*c3.3*	ENSONIP00000000385
	*c3.2*	ENSGMOP00000013815		*c3.4*	ENSONIP00000020700
	*c3.3*	ENSGMOP00000011535		*c5*	ENSONIP00000000818
	*c3.4*	ENSGMOP00000011037	***Oryzias latipes***	*c3.1*	ENSORLP00000014130
***Xiphophorus maculatus***	*c3.1*	ENSXMAP00000012461		*c3.2*	ENSORLP00000022744
	*c3.2*	ENSXMAP00000012489		*c3.3*	ENSORLP00000014189
	*c3.3*	ENSXMAP00000013785		*c3.4*	ENSORLP00000024945
	*c3.4*	ENSXMAP00000003878*		*c5*	ENSORLP00000021960
	*c5*	ENSXMAP00000004243*	***Takifugu rubripes***	*c3.1*	ENSTRUP00000027014
***Tetraodon nigroviridis***	*c3.1*	ENSTNIP00000017117		*c3.2*	ENSTRUP00000045164
	*c3.2*	ENSTNIP00000009066		*c3.3*	ENSTRUP00000007069
	*c3.3*	ENSTNIP00000021226		*c3.4*	ENSTRUP00000004959
	*c3,4*	ENSTNIP00000017050		*c3.6*	ENSTRUP00000006634
	*c5*	ENSTNIP00000008583		*c5*	ENSTRUP00000032286

*Full gene sequence not available.

For *Danio rerio* analysis, GenBank sequences were used (NP_571317.1, NP_571318.1, NP_001032313.1, XP_002660623.2, XP_002660624.2, NP_001008582.3, NP_001093490.1, NP_001093483.1) instead of the Ensembl ones.

To identify gene duplications and loss events during the evolution of the *c3–c5* gene family, a reconciliation [23], [24] of the *c3–c5* tree with fish phylogeny was performed. Although the evolutionary relationships among fish species are not well resolved [25], in this study, a species tree coherent with the accepted taxonomic relationships among teleost species was selected (Figure S1). The divergence times among fish species were retrieved from the TimeTree database [26]. The most parsimonious reconciliation of the estimated gene tree and the species tree was performed with Notung 2.6 [27], [28] and represented with FigTree v1.3.1 (http://tree.bio.ed.ac.uk/software/figtree/) and PrimeTV [29]. Synteny was investigated with Genomicus v69.01 [30].

### Animals

Fish care and challenge experiments were conducted according the CSIC National Committee on Bioethics guidelines under approval number ID 01_09032012.

The wild-type and Tg(mpx:GFP) [31] zebrafish used in this study were obtained from our experimental facility, where they were cultured according to established protocols [32], [33]. Tg(mpx:GFP) fish were kindly provided by S. Renshaw (University of Sheffield).

### Expression of *c3* genes

To evaluate the mRNA expression levels of the *c3* paralogs, total RNA was extracted from several organs collected from naïve wild type adult zebrafish, including the spleen, kidney, liver, intestines, gills, heart, muscle, tail and brain. Samples from 12 fish were pooled to yield 3 biological replicates of 4 individuals per pool.

To determine the expression levels of the different genes during zebrafish ontogeny, wild-type zebrafish larvae were sampled at the following different times post-fertilization (pf): 3 hpf, 6 hpf, 1 dpf, 2 dpf, and 3 dpf and at 3-day intervals from 5 to 29 dpf. Due to differences in animal size, 10–15 animals were necessary to yield biological replicates from 3 hpf to 14 dpf, whereas only 6–8 individuals from 17 to 29 dpf were used for biological replicates. Total RNA was isolated from 3 biological replicates per sampling point.

Furthermore, the post-stimulation expression patterns were analyzed. Adult zebrafish (n = 36) were injected intraperitoneally with 10 µL of 1 mg/mL lipopolysaccharide (LPS) to mimic a bacterial infection. Additional fish (n = 36) were injected with PBS and used as controls. At 3, 6 and 24 h post-stimulation, selected organs (spleen, kidney and liver) were sampled and pooled from 12 fish to yield 3 biological replicates of 4 fish per sampling point per organ.

### Quantitative PCR gene expression analysis

Total RNA isolation was performed for both adults and larvae with the Maxwell 16 LEV simplyRNA Purification kit (Promega, UK). Next, 500 ng of total RNA were used to obtain cDNA with the SuperScript II first-strand synthesis kit and random primers (300 ng/mL; Life Technologies).

*C3* expression patterns were analyzed by quantitative PCR (qPCR). Whenever possible, specific PCR primers for each form were designed with Primer3 software [34]. However, due to their high identity percentage, *c3.2* and *c3.3* were amplified together with common primers and were noted as *c3.2/3.* Similarly, *c3.7* and *c3.8* (*c3.7/8*) were amplified with common primers and analyzed together (Table 2).

**Table 2:** Primer sequences used in this study.

Gene	Function	Primer	Sequence	Amplicon
***c3.1***	qPCR	Forward	TCCAGACAAGCGAAAGGTG	204 bp
		Reverse	CCATCAGTGTACACAGCATCATAC	
***c3.2/3***	qPCR	Forward	CGGTACACAAACACCCCTCT	144 bp
		Reverse	GTCTTCCTCATCGTTCTCTTGTT	
***c3.4***	qPCR	Forward	CAACTCAGAAGCGTCCATGA	145 bp
		Reverse	ATTGATCAGCCCTTGCAACT	
***c3.5***	qPCR	Forward	GTTGCACGCACAGACAAGTT	166 bp
		Reverse	CAGGCTCTTTCTCCATCTGC	
***c3.6***	qPCR	Forward	CAGACCACATCACTGCCAAC	169 bp
		Reverse	TTGTGCATCCGAAGTTGAAG	
***c3.7/8***	qPCR	Forward	CTCCATTTCGATGGCTGAAT	166 bp
		Reverse	ACATCACTCCGACCAGGAAC	
***ef1a***	qPCR	Forward	GCATACATCAAGAAGATCGGC	121 bp
		Reverse	TCTTCCATCCCTTGAACCAG	
***il1b***	qPCR	Forward	TTCCCCAAGTGCTGCTTATT	149 bp
		Reverse	AAGTTAAAACCGCTGTGGTCA	
***c3.1***	ORF amplification	Forward	CTGGAACACAGTCTCGATGG	4958 bp
		Reverse	CAGTAGACAATTATGTTGCACATCC	
***c3.1***	Splicing confirmation	Forward	AAGCTGCAAATAAGCGGAGA	624 bp
		Reverse	GGCTGAGGCTGGACAGTTAT	
***c3.7/8***	Splicing confirmation	Forward	GGTGATGTTGGAGCAAAGGT	762 bp
		Reverse	CCACAACCACGACTCAAAAA	

Primer efficiency was calculated from the slope of the cycle threshold (Ct) regression line versus the relative cDNA concentrations in serial 5-fold dilutions. A melting curve analysis was also performed to verify that no primer dimers were amplified.

Each reaction was performed in 25 µL of reaction mix that comprised 1 µL of 2-fold diluted cDNA template, 0.5 µL of each primer (both at a final concentration of 10 µM; sequences shown in Table 2), 12.5 µL of Brilliant II SyBR Green qPCR Master Mix (Agilent Technologies) and 10.5 µL of pure water. Technical triplicates were performed for each reaction. The cycling conditions were as follows: 95°C for 10 min, 40 cycles of 95°C for 15 s and 60°C for 1 min, and a final dissociation stage of 95°C for 20 s, 60°C for 20 s and 95°C for 20 s. For normalization purposes, the elongation factor 1-alpha (*ef1a*) gene expression levels were analyzed in each sample as a housekeeping gene control according to the Pfaffl method [35]. Additionally, *il1b* mRNA expression was analyzed in the 3 h samples from LPS-stimulated fish and PBS-injected controls to confirm the inflammatory state after stimulation.

### Gene knockdown studies

For gene inhibition studies, four different morpholinos (Gene Tools) were designed: two translation blocking morpholinos (MO-ATG-c3u and MO-ATG-c3.7/8) and two splice-site blocking morpholinos (MO-c3.1s and MO-c3.7/8s). ATG morpholinos were designed according to the first 25 bases of the sequence. Because of their high sequence identity, a common ATG morpholino was designed to block the genes *c3.1*, *c3.2/3* and *c3.6* (MO-ATG-c3u: CAGAGAGAAACAGCAGCTTCACCAT), while a second ATG morpholino (MO-ATG-c3.7/8: CCCATAACAGCAGCTGAAGAAGCAT) was designed to inhibit *c3.7* and *c3.8*. Splice-site blocking morpholinos were designed according to the *Ensembl* gene exon data (MO-c3.1s: CCAGCTTCTCACCCAGTGTTGCCGT; MO-c3.7/8s: TTCCGACTTACCGAGCTGATCTCT). A standard control oligo (Gene Tools) was used as a non-specific control. Intra-yolk microinjections of morpholino solution, 1 nL, were administered to fresh single-cell stage WT embryos. The splice-site blocking morpholinos efficacy was confirmed by electrophoresis gel of PCR products amplified with primers showed in Table 2. Both the *c3.1* inhibiting morpholinos produced an increase in developmental errors that led to higher lethality when injected at concentrations greater than 0.5 mM in a dose-dependent fashion, and thus, 0.4 mM was used in the study; a 1 mM solution of MO-ATG-c3.7/8 and 0.5 mM of MO-c3.7/8s were used. For the *c3.1* phenotype rescue, the *c3.1* ORF was produced by PCR and capped mRNA was synthetized using mMessage mMachine Kit (Ambion) following the manufacturer protocol.

### Neutrophil migration studies

For neutrophil migration studies, morpholino microinjections were performed in Tg(mpx:GFP) zebrafish embryos. At 3 dpf, after larval hatching, the tails were cut with a razor. 24 h Neutrophil migration to the regenerative tissues was observed under an AZ100 microscope (Nikon) and photographed with a DS-Fi1 digital camera (Nikon) and the relative fluorescence intensities in the tails resulting from the GFP expressed under the myeloid-specific peroxidase promoter present in the neutrophils was measured with ImageJ software [36].

Confocal images of 3 h post fin transection live larvae were captured using a TSC SPE confocal microscope (Leica). The images were processed using the LAS-AF (Leica) and ImageJ software.

### Aeromonas hydrophila infection

To evaluate the effect of the *c3.7/8* inhibition on the inflammation, 3 dpf larvae microinjected with the MO-c3.7/8s morpholino were infected with a concentration 3·10^7^ *A. hydrophila* by bath. At 3 h post infection, total RNA from 3 biological replicates of 10 individuals was extracted and analyzed by qPCR.

### In situ hybridization

Sense and antisense RNA-probes were designed according to the previously used qPCR primers. The probes were produced with the PCR amplification method under standard PCR conditions (35 cycles, 60°C annealing temperature). The sense probe incorporated the necessary promoter sequence for labeling purposes (*SP6* promoter, ACGATTTAGGTGACACTATAGAA), while the antisense probe incorporated the *T7* promoter (AGTTAATACGACTCACTATAGGGATT). RNA-probes were prepared with the DIG-RNA Labeling Kit (SP6/T7) (Roche) according to the manufacturer's instructions. Whole-mount in situ hybridization (ISH) was performed on 3 dpf zebrafish embryos essentially as reported by Thisse and Thisse [37]. Stained embryos were cleared in 100% glycerol, observed under an AZ100 microscope (Nikon) and photographed with a DS-Fi1 digital camera (Nikon).

## Results

### Sequence, genomic organization and phylogeny of zebrafish *c3*

Previous screening of the zebrafish genomic PAC library identified three loci for complement *c3*, namely, *c3.1*, *c3.2* and *c3.3* [11]. We sought to extend this analysis by performing a genome-wide blast search. Our analysis not only confirmed the presence of genes coding for the *c3.1*, *c3.2* and *c3.3* variants in the zebrafish chromosome 1 but further identified three putative *c3* genes in chromosome 1 and two in chromosome 22, which were named *c3.4*, *c3.5*, *c3.6*, *c3.7* and *c3.8* (Table 1).

The predicted proteins (C3.1 to C3.6) coded by the *c3* genes located in chromosome 1 showed an overall identity above 55% and a similarity above 70%. In contrast, they only shared 35% identity and 57% similarity with the putative C3 proteins located on chromosome 22 (Table 3). Furthermore, the similarity between human C3 and zebrafish C3.1 to C3.6 was approximately 43%, whereas a slightly lower similarity was observed between the human C3 and zebrafish C3.7 and C3.8 sequences (Table 3). Due to their high similarity, we analyzed *c3.2* and *c3.3* as a group (*c3.2/3*). The same strategy was adopted for *c3.7* and *c3.8* (*c3.7/8*). Additionally, similar conserved domains, such as MG1, A2M, C345C and anaphylatoxin (ANATO), were observed in both human C3 and zebrafish C3, according to the CDD. The MG1 and ANATO protein domains were not initially predicted in the C3.8 protein sequence, but partial cDNA amplification and posterior sequencing confirmed the presence of the latter domain (data not shown).

**Table 3:** Identity (right) and similarity (left) table of the zebrafish, human and mouse C3.

	C3.1	C3.2	C3.3	C3.4	C3.5	C3.6	C3.7	C3.8	HsC3	MmC3
**C3.1**		69.3	69.1	55.2	60.0	61.5	35.5	35.3	43.2	43.2
**C3.2**	83.5		**90.7**	54.1	58.1	58.7	35.0	34.9	43.3	44.2
**C3.3**	83.4	**94.5**		54.2	58.3	58.9	35.2	35.2	43.1	43.4
**C3.4**	75.0	73.1	73.1		55.0	54.9	35.4	34.8	43.1	43.1
**C3.5**	77.5	75.3	75.0	73.3		62.0	36.6	36.0	42.7	43.2
**C3.6**	78.9	77.1	77.5	74.2	78.8		35.4	35.1	41.0	42.1
**C3.7**	59.3	58.9	58.9	58.5	59.3	58.3		**91.1**	37.4	36.9
**C3.8**	58.5	58.0	57.8	57.5	58.3	57.3	**95.1**		36.7	36.5
**HsC3**	64.3	63.1	62.5	64.3	63.5	63.0	58.6	57.5		77.0
**MmC3**	65.1	63.7	63.3	63.9	63.1	63.2	58.0	57.3	88.6	

Accession number for *Homo sapiens* C3: NP_000055.2 and *Mus musculus* C3: NP_033908.2.

**Figure 1:**
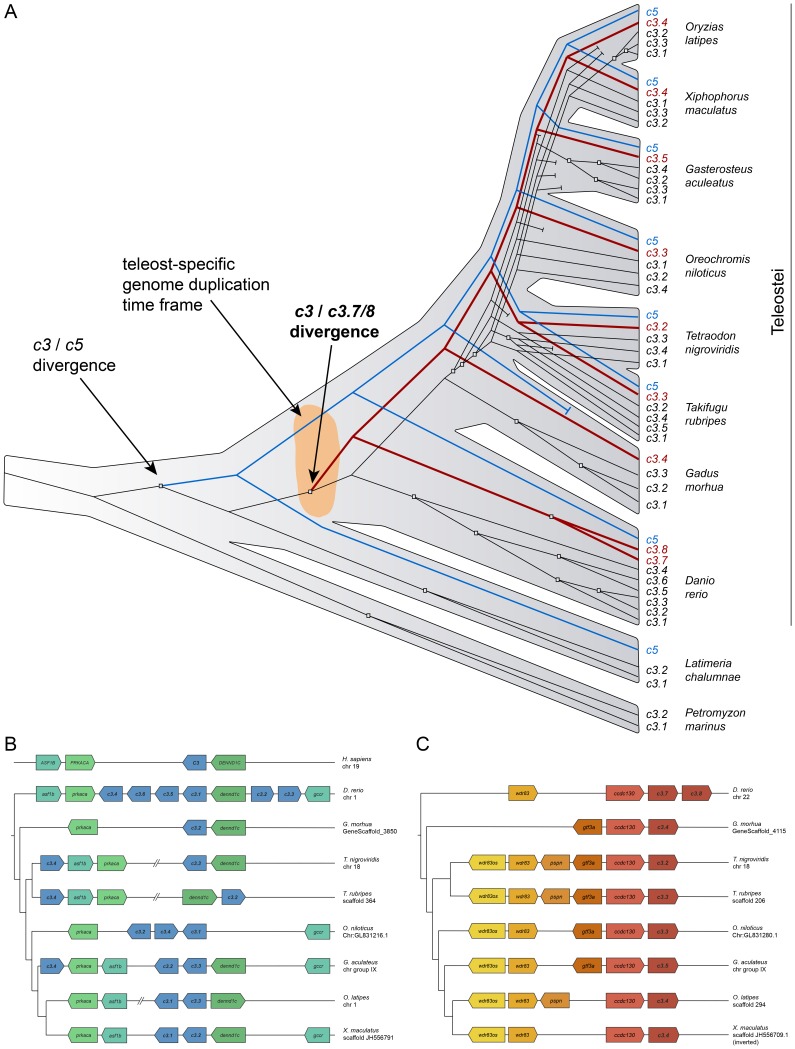
Sequence, genomic organization and phylogeny of zebrafish *c3*. (A) Reconciliation of the *c3* and *c5* gene phylogeny within the evolution of fish species. The blue line follows the *c5* gene tree, while the red line follows the *c3.7/8* gene tree. Duplication events were marked with squares, and losses were marked with perpendicular ends of the gene line. The reconstruction indicated the *C3* and *C5* divergence prior to coelacanth speciation, while the *c3.7/8* divergence was located at approximately the teleost-specific genome duplication time frame (represented in orange). (B) Chromosome 1 *c3* synteny between teleost species studied and *Homo sapiens*. The position and direction of the genes *ASF1B*, *PRKACA* and *DENND1C* that flank the human *C3* gene in the chromosome 19 were conserved and flank the zebrafish genes *c3.1*, *c3.4*, *c3.5* and *c3.6*. The zebrafish *c3.2* and *c3.3* genes were found outside this cluster and in the opposite direction. (C) Conserved chromosome positions between *c3.7/8* and their orthologs across the teleostei species tree. The position and direction of the genes *ccdx130*, *gtf3a*, *pspn*, *wdr83* and *wdr83os* were highly conserved. However, this gene region was inverted in the current platyfish scaffold assembly.

The unreconciled *c3* gene tree was completely resolved, although not all clades were recovered with high confidence (Figure S2). Note, however, that the separation of *c3.7/8* from the other *c3* sequences was highly supported. Interestingly, the reconciled gene tree revealed a highly dynamic gene family with as many as 21 duplications and 10 inferred losses (Figure 1A). In particular, we identified a putative early gene duplication event at the base of the teleostei clade that separated the zebrafish *c3.7* and *c3.8* and their orthologs from the rest of the fish *c3* genes. Indeed, the *c3.7/8* genes seem to be specific to the teleost lineage because a search for orthologs of these genes in non-teleost fish (such as *Latimeria chalumnae* or *Petromyzon marinus*) and other vertebrate genomes returned no results.

Synteny was conserved between teleost *c3* and human *C3* (Figure 1B). The *ASF1B*, *PRKACA* and *DENND1C* genes, which flank the zebrafish *c3.1*, *c3.4*, *c3.5* and *c3.6* genes, are found next to human *C3* on chromosome 19. In addition, the genes had the same orientation. *c3.2* and *c3.3*, however, were placed in the opposite direction and outside this gene cluster next to *DENND1C*. Furthermore, *c3.7/8* synteny was notably well preserved across all tested teleost genomes (Figure 1C).

### Differential expression of the zebrafish *c3* genes

**Figure 2:**
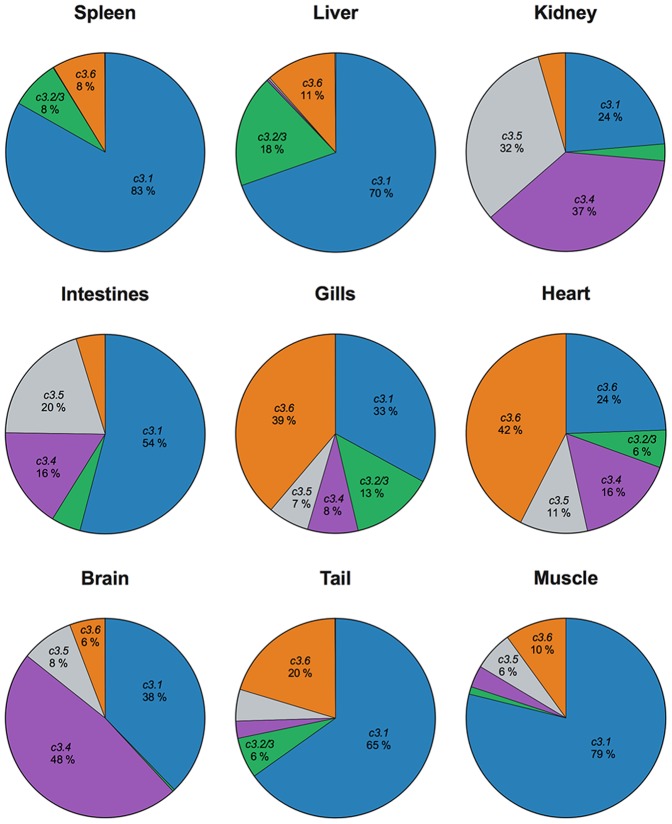
Basal expression of *c3* in adult zebrafish tissues. *c3.1*, *c3.2/3*, *c3.4*, *c3.5*, *c3.6* and *c3.7/8* mRNA expression was evaluated in different adult zebrafish tissues. *c3.7/8* was not represented in the graphics due to its low expression in comparison to the other *c3* genes. The graphs depict the mean results from 3 different experiments, each using a pool of 4 animals.

Next, we evaluated the mRNA expression levels of each *c3* gene in the different zebrafish tissues. All genes were constitutively expressed in the different tested tissues, except for *c3.7/8*, which was not detected in the heart and muscle (Figure 2). Although the expression profile varied in an organ-dependent manner, overall, *c3.1* was the most expressed *c3* gene, followed by *c3.6*. While *c3.1*, *c3.2/3*, *c3.6* and *c3.7/8* followed a similar pattern with predominant expression in the spleen and liver, *c3.4* and *c3.5* were mainly expressed in the kidney and intestine. The *c3.7/8* expression was the lowest, accounting for approximately 1% of the total *c3* expression (Figure 2).

**Figure 3:**
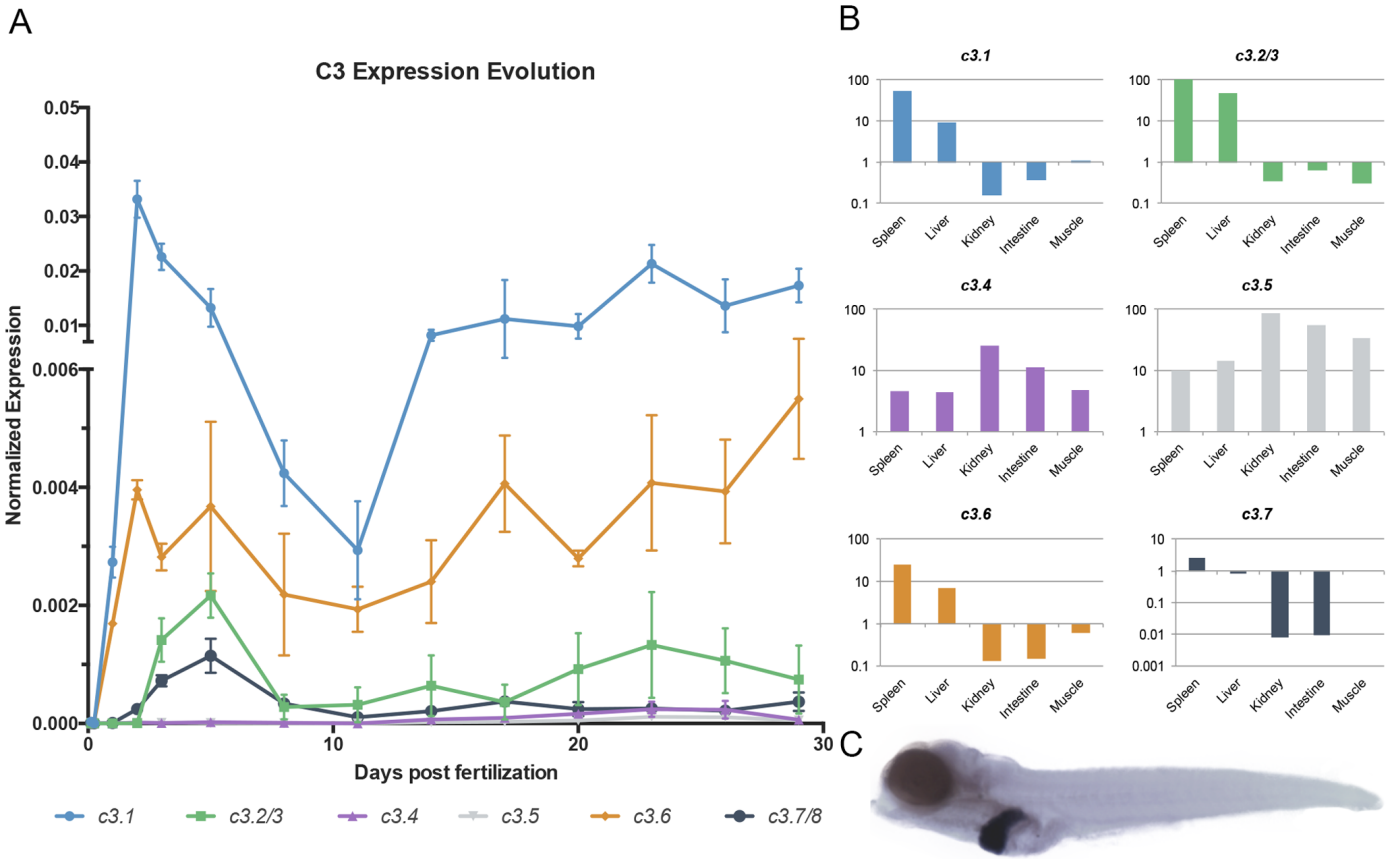
*c3* expression during development. (A) *c3* expression was evaluated in zebrafish larvae over time. *c3* expression values were normalized against the expression of elongation factor 1-α. The graph depicts the mean results from 3 different experiments, each using a pool of 10–15 (3 hpf to 14 dpf) or 6–8 (17 dpf to 29 dpf) animals. (B) *c3* expression relationships between the adult and larval stages. *c3* expression values from adult individuals were divided by the mean values obtained during larval ontogeny. (C). In situ hybridization of a 5 dpf zebrafish larvae showing primary *c3.1* expression in the liver.

During ontogeny, all of the different genes were expressed through larval development with the exception of *c3.2/3*, which was first detected at 48 h of development (Figure 3A). As in the adult tissues, *c3.1* and *c3.6* were the most highly expressed sequences in the larval stages (Figure 3A). Notably, *c3.4* and *c3.5* expression seemed to be predominant in the adult stage, independent of the tissue. In contrast *c3.1*, *c3.2/3*, *c3.6* and *c3.7/8* expression was more prominent during the larval stages in the kidney, intestine and muscle tissues (Figure 3B), possibly indicating a role for these genes in the kidney, intestine and muscle during development. Furthermore, a zebrafish whole-mount *in situ* hybridization confirmed the primary hepatic expression of *c3.1* during development (Figure 3C).

**Figure 4:**
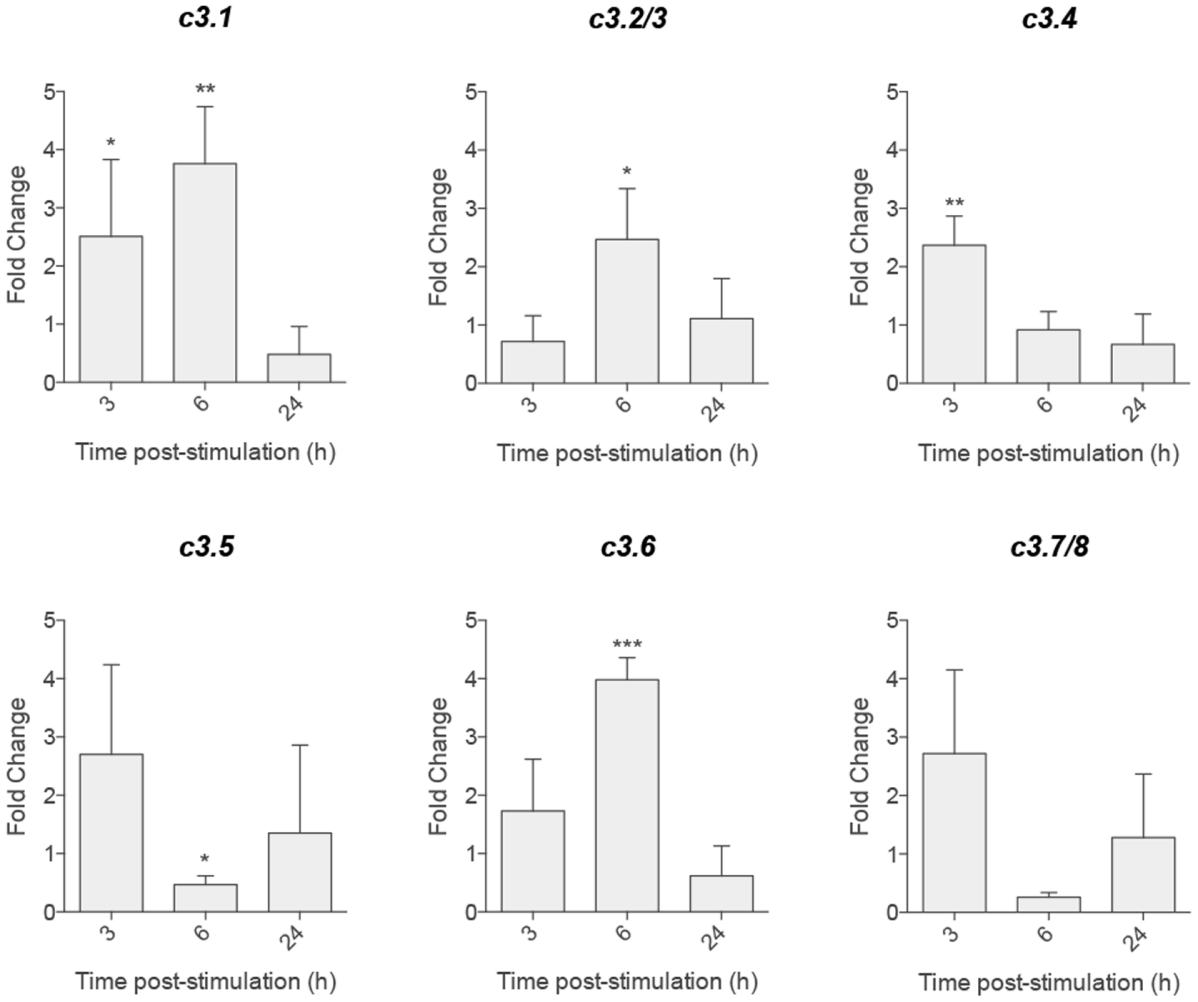
*c3* Expression in response to an inflammatory stimulus. Expression of *c3* was evaluated over time in the spleens of adult zebrafish upon intraperitoneal stimulation with LPS (10 µL of a 1 mg/mL solution). N = 4 animals per group. Statistical significance was determined by independent unpaired T-tests for each time point relative to the non-stimulated sample (*P<0.05, **P<0.01, ***P<0.001).

To investigate whether the induction of *c3* expression was dependent on the *c3* gene, zebrafish were stimulated with LPS, and the *c3* expression levels in the spleen, kidney and liver were subsequently evaluated over a period of 24 h post-stimulation. While an overall increase in the splenic *c3* expression levels was observed upon LPS stimulation (Figure 4), no significant alterations in *c3* expression were found in the kidney or liver (data not shown). The *c3.1*, *c3.2/3* and *c3.6* expression levels peaked at 6 h and demonstrated a significant 3- to 4-fold increase relative to the PBS control. In contrast, splenic *c3.4*, *c3.5* and *c3.7/8* responded early to the treatment with an expression peak at 3 h post-stimulation (Figure 4). In addition to the increased *c3* expression, *il1b* mRNA levels were also increased (22-fold in the spleen after 3 h of LPS stimulation), thus confirming the induction of a pro-inflammatory state by LPS (data not shown).

### Zebrafish *c3* genes possess differential inflammatory roles

**Figure 5:**
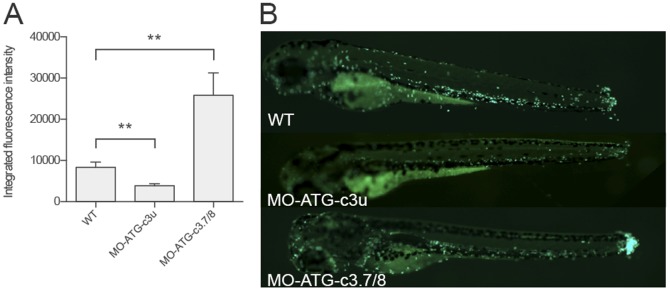
Zebrafish *c3* inhibition results in differential abilities to induce neutrophil migration. The tails of Tg(mpx:GFP) zebrafish were amputated from 3 dpf individuals in which the *c3.1-2/3-6* or *c3.7/8* had been inhibited with the MO-ATG-*c3u* or MO-ATG-*c3.7/8* morpholinos, respectively, and were visualized after 24 h. (A) Relative fluorescence intensities in the amputated zones were calculated with ImageJ (arbitrary units). N = 20 animals per group. Statistical significance was determined by unpaired T-tests (**P<0.01). (B) Fluorescence microscopy images of the treated phenotypes show differential neutrophil migration activities. The figure depicts a representative of 3 experiments.

Because a pro-inflammatory phenotype in zebrafish was associated with the increased expression of all genes (Figure 4), we investigated how the different *c3* affected the migratory abilities of neutrophils in response to tail amputation. To this end, tails of GFP-transgenic [Tg (mpx:GFP)] zebrafish were amputated in individuals in which the *c3.1*-*2/3*-*6* or *c3.7/8* genes had been inhibited with the MO-ATG-c3u and MO-ATG-c3.7/8 morpholinos, respectively. 24 h after the tail injuries, migrating neutrophil estimations were determined by fluorescence microscopy (Figure 5A, B). Interestingly, while inhibition of *c3.1*-*2/3*-*6* resulted in a 2-fold decrease in neutrophil migration to the mutilated zone, the inhibition of *c3.7/8* had an opposite effect with a 2-fold increase in the number of migrating neutrophils in the damaged tissue, suggesting that the different *c3* genes have opposite roles during the inflammation process in zebrafish.

**Figure 6:**
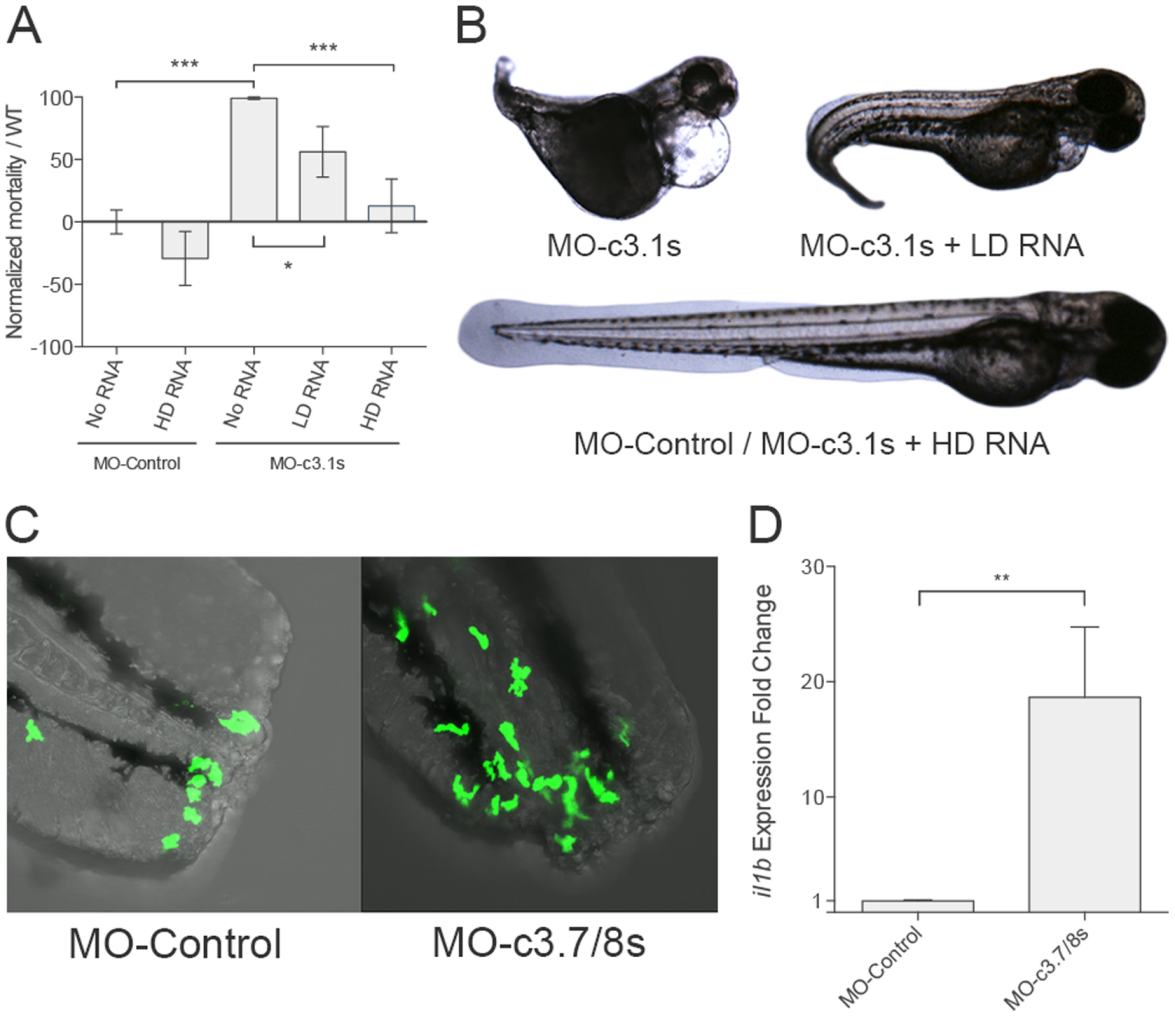
Developmental and inflammatory effects of the *c3.1* and *c3.7/8* inhibition. (A) MO-c3.1s injected at high concentrations produced aberrant phenotypes and increased mortality. Co-injecting with *c3.1* capped mRNA recovered the WT phenotype in a dose-dependent manner (LD: 2,25 ng *c3.1* capped RNA; HD: 4,5 ng *c3.1* capped RNA). (B) Representative individuals of the *c3.1* inhibition treatments and its phenotypic rescue. (C) MO-c3.7/8s microinjected larvae showed a major migration number of neutrophils to the amputation site 3 h after tail clipping (confocal images). Statistical significance was determined by unpaired T-tests (**P<0.01, ***P<0.001). (D) MO-c3.7/8s microinjected larvae significantly expressed higher levels of *il1b* 3 h after bath infection with *A. hydrophila*.

These results for *c3.1* and *c3.7/8* inhibition were confirmed using splice-site blocking morpholinos (Figure 6). Similar to MO-ATG-C3u, MO-c3.1s affected the development in a concentration-dependent manner. However in this case, stronger effects were observed since the minimal concentration that successfully blocked the *c3.1* expression affected the larvae phenotype. In consequence, it was not possible to determine its effects on neutrophil migration. The co-injection of the morpholino with *c3.1* capped mRNA successfully rescued the aberrant phenotypes (Figure 6A, B).

The increased inflammatory state observed with the inhibition of *c3.7/8* ATG morpholino was also successfully confirmed with the splice-site blocking morpholino MO-c3.7/8s. Neutrophil migration studies after tail clipping was higher in the MO-c3.7/8s injected individuals (Figure 6C). Unfortunately, in this case the synthesis of capped RNA was not successful, probably due to the length of the gene. Moreover, *il1b* expression was found to be significantly higher in MO-c3.7/8s microinjected larvae than on WT 3 h after its stimulation with *A. hydrophila* bath infection (Figure 6D).

## Discussion

Herein, we characterized eight genes that code for the C3 protein in zebrafish (*c3.1* to *c3.8*). The first three genes, *c3.1*, *c3.2* and *c3.3*, also known as the *c3a*, *c3b* and *c3c* genes, have been previously identified [11]. However, *c3.4* to *c3.8* sequences were present in zebrafish online databases like NCBI and Ensembl but lacked of proper characterization. Notably, multiple forms of complement components, such as C3, C5, C7, factor B and properdin, have been identified in lower vertebrates [8], [38]–[40], raising the hypothesis that this remarkable diversity has allowed these animals to expand their innate capacities for immune recognition and response [8].

The phylogenetic analysis of the different *c3* and *c5* genes suggests a high genomic dynamism in teleostei as multiple copies of the *c3* gene were observed in all analyzed fish genomes (Figure 1A). In contrast, the evolution of the *c5* gene family was much more static. Here, the absence of the *c5* gene in lampreys correlates well with the hypothesis that this gene appeared in the jawed fish lineage [41]. Thus, *P. marinus c3*, instead of the *c3*/*c5* duplication event, was used to root the phylogenetic tree.

Our analysis also indicates that the *c3.1* to *c3.6* genes in zebrafish and the majority of the *c3* genes in medaka, stickleback and cod resulted from intraspecific duplications of a unique ancestral *c3* gene. Additionally, a particular *c3* gene duplication that was conserved across the analyzed genomes appeared in the teleost lineage, resulting in the *c3.7* and *c3.8* genes and their orthologs. This particular paralog *c3* gene seems to be specific to teleosts, as indicated by the fact that we could not locate orthologs of this gene in non-teleost fish (coelacanth and lamprey) or other vertebrate genomes.

The *c3.1* to *c3.6* genes are found in tandem in the zebrafish chromosome 1. This particular gene order could indicate that these genes are the products of specific, segmental duplications and not the consequence of whole genome duplications and posterior rearrangements, a hypothesis that agrees with the inferred gene phylogeny. The conserved synteny between the zebrafish chromosome 1 *c3* genes and human *C3* indicates that those genes are likely orthologs and are therefore expected to retain equivalent functions [42]. In contrast, *c3.7* and *c3.8* are found in chromosome 22 and, although we did not specifically study their origins, could have possibly emerged during teleost-specific genome duplication. Regardless, we can safely state that the *c3.7/8* orthologs demonstrate a different evolutionary pattern than the other *c3* duplications.

It is necessary to indicate that we worked with draft genomes, for which the assemblies and annotations are incomplete. This is important to remember when drawing conclusions from the analyses. For example, the disappearance of the *c5* gene in Atlantic cod is likely an artifact of an unfinished genome assembly rather than a real gene loss. Additionally, *c3.5* appears as two distinct gene products in the current zebrafish genome and *c3.8* was not correctly predicted, thus positioning the anaphylatoxin domain inside a non-existent intron.

Gene expression analysis revealed two different expression patterns in the studied tissues, which included the spleen, liver, kidney, intestine, gills, heart, brain, tail and muscle. *c3.1*, *c3.2/3*, *c3.6* and *c3.7/8* were primarily expressed in the spleen and liver. In contrast, *c3.4* and *c3.5* showed higher expression in the kidney and the intestine. This expression pattern contradicts that observed in mammals, in which complement factors are mainly secreted in the liver. However, high extrahepatic *c3.2* and *c3.3* expression was also observed in fish in response to Poly I:C [43] and viruses [44], suggesting the local production of innate immune proteins in response to infection. As expected, the *c3* genes were highly and early expressed during ontogeny, when adaptive immunity is not yet developed. A similar pattern has been reported in other fish species, such as the India major carp, Atlantic cod, spotted wolffish and Atlantic salmon [45]–[48]. This early expression was primarily located in the liver as determined by *in-situ* hybridization of *c3.1* on 5 dpf zebrafish larvae, agreeing with the data deposited in ZFIN database [49]. *c3.2/3* did not follow this earlier expression pattern and was not detected until 2 dpf, supporting previous findings of low zebrafish *c3.2/3* expression before hatching [50].

In addition to constitutive expression, LPS-induced expression also revealed the differential regulation of *c3* in the different organs. While the splenic expression levels of *c3.1*, *c3.2/3* and *c3.6* reached the maximum increase of 3-4-fold at 6 h post-stimulation, *c3.4* and *c3.5* only showed early (3 h post-stimulation) increased splenic expression. These values are similar to the *c3.1* levels reported for *M. marinum*-infected zebrafish [51]. The paralog *c3.7/8* only showed early incremental expression after LPS treatment. In agreement with the differential expression, we also detected differential abilities of the *c3* paralogs to modulate the developmental and inflammatory process.

On the one hand, a strong inhibition of *c3.1* using morpholinos resulted in an increased percentage of aberrant phenotypes, such as an increased presence of edemas and difficulties in yolk sac resorption, which derived in non-hatched individuals. Co-injection of *c3.1* mRNA successfully rescued the phenotype in a dose-dependent style. The *c3.1* anaphylatoxin fragment (C3.1a) has already been shown to control mutual cell attraction during chemotaxis [52] and been correlated with the tissue regeneration process in zebrafish [53] as well as other species [54], [55]. Furthermore, a mild, partial inhibition of c*3.1*, *c3.2/3* and *c3.6* resulted in diminished neutrophil migration to the injury site.

On the other hand, inhibition of *c3.7/8* did not affect the zebrafish development, but significantly altered the magnitude of the response after inflammatory stimuli. Fish microinjected with *c3.7/8* morpholinos showed a great fold-change increase of proinflammatory *il1b* cytokine expression 3 h after *A. hydrophila* infection as well as massive neutrophil migration to the regenerative tissue after tail clipping at both 3 and 24 h. This suggests that *c3.7/8* plays an important role in complement regulation and inflammation modulation. In summary, our results show that *c3.7/8* is a paralog *c3* gene found exclusively in teleost fish; this paralog has the same structure as classical *C3* but might regulate inflammatory responses to maintain an optimal equilibrium between reactions against external stimuli and protection against cell damage.

## Supporting Information

Figure S1 **The species tree used in this study was coherent with the current taxonomic information.**
(TIF)

Figure S2 **The unreconciled C3–C5 tree confidently separated the different C3–7/8 of the rest of C3 and C5 sequences.** Abbreviations used: Dr – *Danio rerio*, Gm – *Gadus morhua*, Ga – *Gasterosteus aculeatus*, Ol – *Oryzias latipes*, Xm – *Xiphophorus maculatus*, On – *Oreochromis niloticus*, Tr – *Takifugu rubripes*, Tn – *Tetraodon nigroviridis*, Lc – *Latimeria chalumnae*, Pm – *Petromyzon marinus*.
(JPEG)
