# Supplementary figures and images for: The Evolution and Appearance of C3 Duplications in Fish Originate an Exclusive Teleost c3 Gene Form with Anti-Inflammatory Activity
Source: PLoS One. 2014 Jun 13;9(6):e99673. doi: 10.1371/journal.pone.0099673 (PMC4057122; doi:10.1371/journal.pone.0099673)

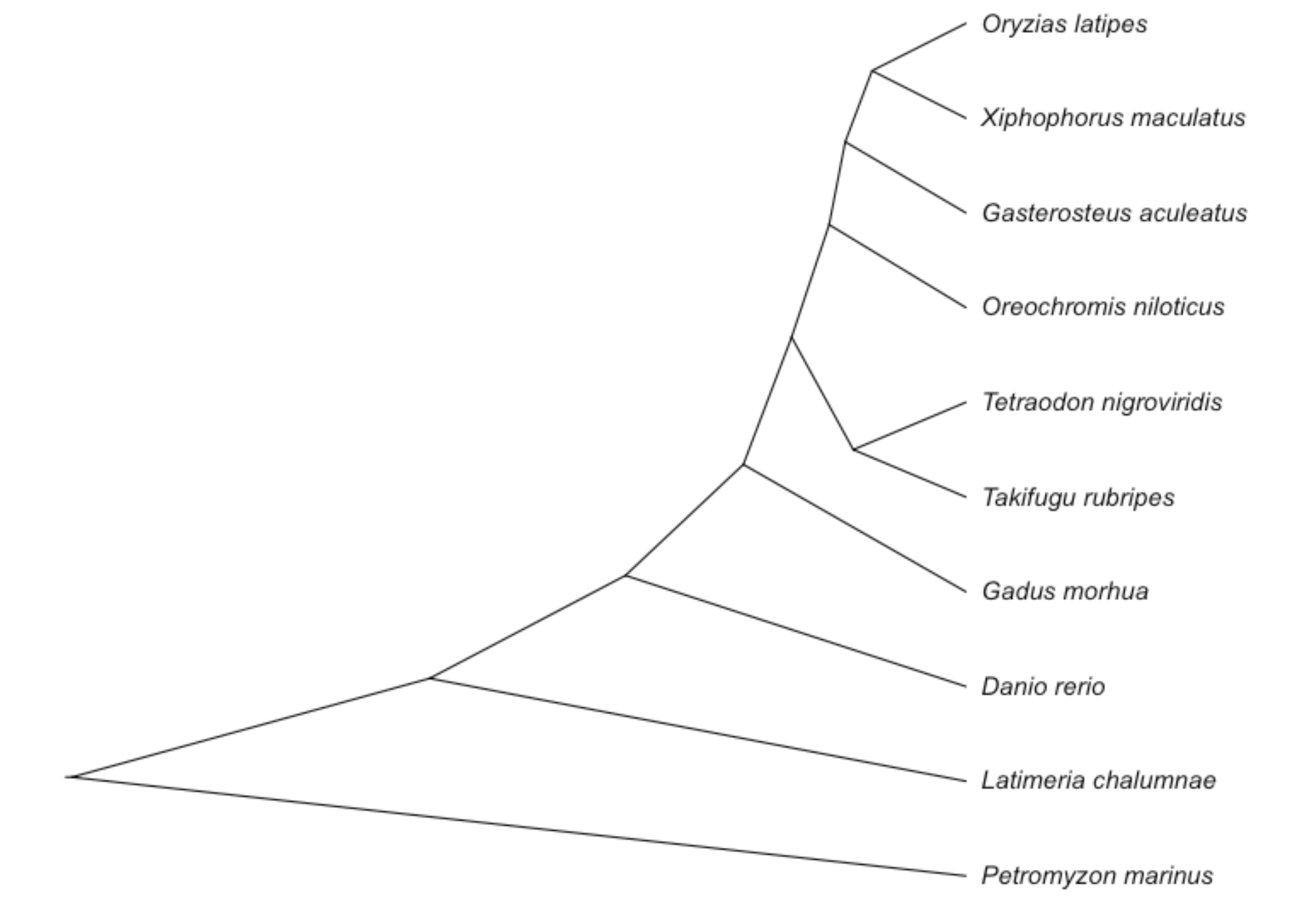

Supplement: Figure S1 — The species tree used in this study was coherent with the current taxonomic information. (TIF) [file pone.0099673.s001.tif]

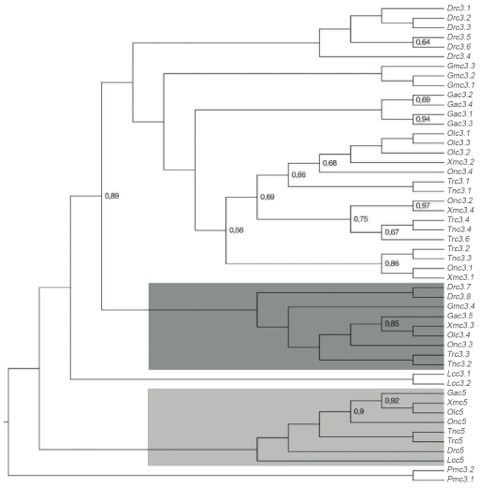

Supplement: Figure S2 — The unreconciled C3–C5 tree confidently separated the different C3–7/8 of the rest of C3 and C5 sequences. Abbreviations used: Dr – Danio rerio, Gm – Gadus morhua, Ga – Gasterosteus aculeatus, Ol – Oryzias latipes, Xm – Xiphophorus maculatus, On – Oreochromis niloticus, Tr – Takifugu rubripes, Tn – Tetraodon nigroviridis, Lc – Latimeria chalumnae, Pm – Petromyzon marinus. (JPEG) [file pone.0099673.s002.jpeg]
